# ORAL MANIFESTATIONS IN HUMAN IMMUNODEFICIENCY VIRUS INFECTED PATIENTS

**DOI:** 10.4103/0019-5154.60369

**Published:** 2010

**Authors:** Sumit Sen, Sukanta Mandal, Sourav Bhattacharya, Saswati Halder, Parna Bhaumik

**Affiliations:** *From the Department of Dermatology, Venereology and Leprology, North Bengal Medical College and Hospital and North Bengal Dental College.*

**Keywords:** *Oral candidiasis*, *seropositives*, *aphthae*

## Abstract

**Background:**

Oral lesions are common among Human Immuno deficiency Virus (HIV)-positive patients. The pattern of oral features in these persons may differ in separate settings.

**Aims:**

To find out the spectrum of oral manifestations among a section of seropositive individuals attending the antiretroviral therapy (ART) centre of a referral hospital.

**Materials and Methods:**

A total of three hundred and twenty-one newly diagnosed seropositive patients were enrolled in this study. Of these, ninety-four patients who demonstrated lesions related to the oral cavity were examined intra-orally by a clinician. HIV testing was done with ELISA and CD4 counts were measured with the help of fluorescence activated cell sorter (FACS) system.

**Results:**

Fifty-four respondents presented exclusively with oral lesions. Candidiasis patients were the largest group (38.30%).

**Conclusion:**

HIV disease presents a plethora of oral manifestations, which provide valuable diagnostic and prognostic information.

## Introduction

All HIV-positive patients are susceptible to oral lesions at some point of their illness.[1] Many such HIV-associated diseases occur initially, thus providing an opportunity for early diagnosis.

The oral presentations reflect the underlying immune status of the patient. Morphology and multiplicity of these lesions in an individual are correlated with disease progression. A CD4^+^ lymphocyte count of less than 200/mm^3^ has been postulated as indicative of active disease with early progression to death.[2,3] Therefore, the CD4^+^ count can be used to monitor the progress of the disease and plan suitable therapy.

The purpose of this study was to explore the oral features of a group of patients and survey their CD4^+^ counts.

## Materials and Methods

A total of three hundred and twenty-one complainants attending the Anti-Retroviral Therapy (ART) centre, newly tested positive for HIV from December 2007 to September 2008, were examined for oral lesions. Each adult patient and the parents of the pediatric patients were quizzed to seek relevant etiological information. Children below eighteen months of age were not included. Cases with multiple disorders were included in the study. Cutaneous findings were also noted. Excision biopsies of the oral lesions were performed by a dental surgeon, where necessary. Suspected cases of oral candidiasis were scraped and cultured on Sabouraud-agar media. HIV testing was done using the Elisa Technique and the seropositives were subjected to CD4^+^ cell counts using the fluorescent activated cell sorter (FACS) count system, based on flow cytometry.

## Results

Ninety-four patients presented with oral manifestations with cutaneous lesions. Fifty-four persons presented with only oral lesions.

A total of fifty-eight males and thirty-six females participated in this study. Most of the affected persons were in the >25-35 years (47.9%) age group and there was no member in the 5-15 years age range [Table 1]. Heterosexual spread was the most common mode of transmission (88.30%) (83 persons) and only four patients admitted to having homosexual relations among these ninety-four persons. One was an intravenous drug abuser. Blood transfusion was the possible cause in an aged spinster who did not give any history of exposure.

**Table 1:** Distribution of HIV-positive patients presenting with oral manifestation according to their age

Age groups (in years)	Frequency	Percent (%)
<5	5	5.3
5-15	0	0
>15-25	13	13.8
>25-35	45	47.9
>35-45	26	27.7
>45	5	5.3
Total	94	100.0

Thirty-six cases (38.30%) of oral candidiasis formed the largest group [Table 2]. Pseudo membranous type of candidiasis was the predominant type of oral candidiasis accounting for 30 of the cases. Angular cheilitis was reported in four instances and hyper plastic and atrophic candidiasis was reported only in single patients. The tongue was the most commonly involved site in our study. Oral candidiasis group presented with mean CD4^+^ count of 212.61/mm^3^ [Table 2]. The lateral sides of the tongue were predominantly affected in all cases of oral hairy leukoplakia and they presented with the lowest mean CD4^+^ cell count (97.89/mm^3^) [Table 2]. Kaposi's sarcoma was noted by its absence. Oral candidiasis was the predominant lesion among the group under five years of age (4 cases). There was a single case of recurrent herpetic ulcer in a child. A total of 22 patients (23.4%) had solitary lesion, 46 individuals (48.9%) had two lesions while the rest 26 had more than two lesions.

**Table 2:** Distribution of study population according to their oral manifestation and Mean CD4 cell counts

Oral manifestation	Number (N)	Percentage (%)	MeanCD4 count
Oral candidiasis	36	38.3	212.61
Aphthous ulceration	13	13.8	157.33
Cervical lymphadenopathy	10	10.6	168.70
Herpes simplex	10	10.6	255.60
Oral hairy leukoplakia	9	9.6	97.89
Linear gingival erythema (LGE)	6	6.4	183.00
Oral pigmentation	4	4.3	286.25
Oral ulcer	3	3.2	215.69
Cheilitis (Non candidal)	2	2.1	144.50
Warty growth	1	1.1	146.00
Total	94	100.0	

## Discussion

The oral health status of a HIV-infected patient at presentation is an extremely important parameter, as it may reveal important information regarding the immune status of the individual.

Oral disorders occur in about 64% cases of HIV/AIDS in India[4,5] and have a prevalence of 56% in the West.[6] The most proficient age group is affected in the developing world. The increasing number of females being affected (male: female = 1.6:1) as reflected in our data is supported by predominant heterosexual mode of transmission and is in stark contrast to another study[7] where males outnumbered females due to large number of males having sex with males. The most common HIV related oral disorder is oral candidiasis which occurs in 17-43% cases with HIV infection and in more than 90% of cases with AIDS.[8] Four types of oral candidiasis are found clinically—atrophic, pseudomembranous, hyperplastic, or chronic and angular cheilitis. While all forms of oral candidiasis are associated with low CD4^+^ cell count, the pseudomembranous variety is correlated with progressive immune deterioration with CD4^+^ counts less than 200 cells/mm^3^.[9] Hodgson's[10] findings support our hypothesis that oral candidiasis is associated with immune suppression and thus has significant prognostic value. Aphthous ulcers, which were found in 13.2% of the population in our study, were in contrast to a Chinese study[11] where the figure was double. Major aphthae occur due to immune dysregulation in seropositives.

The lesions of recurrent oral herpes simplex in HIV-infected persons present with ulceration and pain of longer duration, which can result in reduced intake of food and resultant weight loss, which worsen the morbid condition.

Oral hairy leukoplakia (OHL) is usually caused by Epstein-Barr virus. Studies[12] from the West found higher number of cases of OHL. Such cases have been rarely reported in Indian studies.[4] The larger number of cases from the West may be due to greater proportion of men practising homosexuality. Significantly, OHL is associated with late stage of HIV infection in individuals with CD4^+^ count of less than 200/mm^3^.[3]

There were no cases of Kaposi's sarcoma (KS), possibly due to low number of cases reporting homosexual behavior in contrast to reports from Europe and the United States.[1] Homosexual behavior has been identified as a risk factor for KS. Necrotizing ulcerative periodontitis is associated with CD4^+^ counts less than 200 cells/mm^3^. In the present study, it was not observed due to less number of men having sex with men.

Prepubertal and adolescent children did not present in our group. Pseudomembranous candidiasis was the most frequent oral disorder and OHL presented in advanced HIV disease.
